# Violence typologies and sociodemographic correlates in South African adolescents: a three-wave cross-sectional study

**DOI:** 10.1186/s12889-020-8332-6

**Published:** 2020-02-12

**Authors:** Xincheng Sui, Karlijn Massar, Robert A. C. Ruiter, Priscilla S. Reddy

**Affiliations:** 1 Department of Work and Social Psychology, Faculty of Psychology and Neuroscience, Maastricht University, P.O. Box 616, 6200 MD Maastricht, The Netherlands; 2 Human Sciences Research Council, Cape Town, 8001 South Africa; 3 Visiting Professor, Nelson Mandela University, Port Elizabeth, 6031 South Africa

**Keywords:** Violence, Victimisation, Perpetration, Adolescents, Trends, Sociodemographics

## Abstract

**Background:**

Violence victimisation and violence perpetration may co-occur in adolescents. Understanding the sociodemographic correlates of the independent and joint profiles of victimisation and perpetration may inform preventive interventions. This study examined the associations of sociodemographic factors with four violence typologies, namely, 1) non-involvement in both victimisation and perpetration, 2) victims only, 3) perpetrators only, and 4) victim-perpetrators. Trends in the prevalence of the four violence typologies over the three survey years were also examined.

**Methods:**

We used data from the three nationally representative South African Youth Risk Behaviour Surveys conducted in 2002, 2008, and 2011 and included a multi-ethnic sample of adolescents (*n* = 30,007; boy: 46.9%, girls: 53.1%; M age = 16 years, SD = .06).

**Results:**

The sample consisted of 8030 (30.8%) adolescents who had non-involvement in both victimisation and perpetration, 8217 were victims only (29.8%), 2504 were perpetrators only (9.0%), and 7776 were victim-perpetrators (24.6%). Logistic regression analyses showed that being a girl increased the odds of non-involvement (OR: 1.47, 99% CI: 1.36–1.58) and being victims only (OR: 1.90, 99% CI: 1.76–2.05). Being a boy increased the odds of being perpetrators only (OR: 0.42, 99% CI: 0.37–0.47) and victim-perpetrators (OR: 0.51, 99% CI: 0.47–0.55). Adolescents who did not have an absent mother had higher odds of non-involvement (OR: 0.78, 99% CI: 0.62–0.97). Lower monthly allowance increased the odds of victimisation only (OR: 0.99, 99% CI: 0.97–1.00), whereas higher monthly allowance increased the odds of perpetration only (OR: 1.05, 99% CI: 1.03–1.08). Trend analysis showed that between 2002 to 2011, there was an increase in the prevalence of non-involvement in adolescents (*p* < .001), a decrease in the prevalence of victims only (*p* < .05) and victim-perpetrators (*p* < .001), and no changes in the prevalence of perpetrators only (*p* > .05).

**Conclusions:**

Sociodemographic factors are uniquely associated with different violence typologies suggesting the need for tailored interventions to target adolescents with differed risks to violence victimisation and perpetration. Strengthening family relations, particularly between mother and child, may protect adolescents from the experiences of victimisation and perpetration.

## Background

Globally, violence is a significant public health concern and it is one of the major causes of death among adolescents [1]. Violence can be encountered in various ways, such as directly - through personal experiences of victimisation - or indirectly, through witnessing or hearing about violence [2]. On the other hand, violence can also be perpetrated by intentional uses of physical force to threaten or harm others [3]. In South Africa, violence victimisation and perpetration continue to affect the daily lives of adolescents. They are exposed to violence in many or all of the major developmental contexts including home, school, and community [4, 5]. Moreover, many adolescents experience poly-victimisation (i.e. exposure to multiple and different types of violence) across these contexts [4, 6, 7]. With respect to perpetration, violent behaviours such as bullying peers, engaging in physical fights, and carrying weapons have persisted in South African adolescents over the past decade [8]. Both violence victimisation and perpetration have adverse implications for adolescent developmental outcomes, such as psychological problems and risk behaviours [5, 7, 9], and importantly, these problems may persist into adulthood, affecting their psychosocial functioning and health [10, 11]. Therefore, the current research aims to investigate trends among adolescent violence victimisation and perpetration, and their risk factor correlates to better inform targeted youth interventions in South Africa.

### Risk factors for victimisation and perpetration

Not all adolescents are at risk for violence victimisation and perpetration – one’s developmental outcome is an interplay of both individual characteristics and environmental risk factors [12]. As such, the understanding of the unique patterns of risk factors that are associated with violence victimisation and perpetration among adolescents may be useful for preventive efforts, as it may help predict the likelihood of the negative outcomes occurring and offer opportunities for interventions to combat such negative consequences. Several sociodemographic factors have been found to put adolescents at risk for violence victimisation and perpetration. For example, older adolescents and ethnic minorities are at higher risk for violence exposure [13–16]. Research showed that in a group of South African adolescents from communities of lower socioeconomic status, the majority have encountered violence at home, in school, and in community [4]. Male adolescents are more likely than their female counterparts to encounter violence victimisation in school and community [7], or engage in aggressive behaviours and violence perpetration [17–19].

Furthermore, adolescents’ social context may influence their violence victimisation and perpetration experiences. Specifically, school is one of the major developmental domains where adolescents gather on a regular basis, and this environment can encourage or hamper a range of social and emotional developments [20]. Academic performance in particular reflects an adolescent’s ability to learn and their connectedness with the school. Research found that lower academic performance, for example lack of commitment to school work, poor study skills, and lower grades are consistently associated with a higher risk for involvement in violence [21–24] as well as peer victimisation [25].

Moreover, one’s family structure, particularly residing in a single-parent household is significantly associated with both violence victimisation and perpetration in youth [13, 17]. In South Africa, partly due to the HIV/AIDS epidemic, approximately 3.5 million children are orphaned [26]. One review showed that orphans in sub-Saharan Africa living in extended families are particularly vulnerable to psychological, sexual, and physical abuse [27]. Children from families in which one or more parents are absent typically experience stigma, discrimination, and a general lack of social support [27, 28]. Moreover they have an increased risk for experiencing multiple life adversities and poly-victimisations [13, 29, 30], as well as incarceration later on in life [31, 32].

### Overlap between victimisation and perpetration

Although some studies have examined violence from the perspectives of victimisation and perpetration separately, and provided insight into the risk factors of these types of violence [4, 5, 9], it is important to recognise the extent to which adolescents have been *both* victims and perpetrators of violence. Research has shown that violence victimisation and perpetration are often not distinct experiences and can occur in the same individuals [18, 33]. For example, in a sample of South African children, over half of them had experienced all three types of violence, namely, witnessing violence, violence victimisation, and violence perpetration, in both school and community [5]. Witnessing violence and personal victimisation have also been found to contribute directly to violent behaviours in South African adolescents [34]. Moreover, Jeong et al. [35] found that youth who were victims of school bullying were significantly more likely to have committed a violent offence. Further, there is evidence that violence exposure in childhood (e.g., witnessing parental intimate partner violence, physical abuse) may increase one’s risk for bullying perpetration and peer victimisation [36], as well as perpetration of violence towards partners in intimate relationships as adolescents or adults [37–39]. Therefore, the understanding of violence *profiles* may be limited if victimisation and perpetration are examined separately, since the victimisation group is likely to contain individuals who are also perpetrators, and vice versa [38]. This also means that the sociodemographic correlates of victimisation and perpetration among adolescents may not be clearly unravelled and understood without distinguishing the subgroup of victim-perpetrators from the sample, as victims only, perpetrators only, and victim-perpetrators may each manifest in a unique interplay of individual and environmental factors. For example, a study that examined the independent and joint relationships of victimisation and perpetration in adolescents found that all three violence-affected subgroups were associated with elevated levels of risk factors than the adolescents who had neither victimisation nor perpetration experiences. Furthermore, there are variations in the risk profiles across the violence subgroups as victim-perpetrators demonstrated the greatest psychosocial impairment, such as lower family finances, single parent family, higher number of life stressors, and higher levels of risk behaviours [40].

In addition, studies found that adolescents who are both victims and perpetrators of violence are at higher risk for adverse developmental outcomes, such as socially deviant behaviours, peer rejection [41], depression, and alcohol and drug use [18, 42]. These findings suggest that victim-perpetrators are especially vulnerable to maladjustment, pointing to the importance to include this subgroup of adolescents in research to discern commonalities and distinctions in the associated correlates in comparison with other violence typologies.

### This study

Although violence is recognised as a significant public health concern in South Africa, the prevalence of youth violence victimisation, violence perpetration, and the overlap of the two have received little attention. One aim of the current research is to examine the psychosocial correlates associated with different subgroups of adolescents affected by violence, by considering both the distinctive and joint nature of victimisation and perpetration. Specifically, we consider the four violence typologies that have been consistently documented in the literature, namely, 1) neither victimisation nor perpetration histories, 2) victimisation histories only, 3) perpetration histories only, and 4) both victimisation and perpetration histories [15, 17, 18, 38, 40]. Differentiating violence as such can offer a more comprehensive understanding of the unique correlates of each violence typology [17, 40], which in turn enables tailored intervention development. In the current study, we perform secondary data analysis on three cross-sectional surveys that were conducted in a nationally representative sample of South African adolescents in 2002, 2008, and 2011, with the following aims: 1) to examine the prevalence of the four different violence typologies and their sociodemographic correlates at each survey time point; 2) to examine the associations of demographics, academic performance, and parental absence with each violence typology; 3) to establish trend changes in the prevalence of the four violence typologies from 2002 to 2011.

## Methods

### Participants

This study used secondary data from three cross-sectional South African Youth Risk Behaviour Surveys (YRBS) conducted in 2002, 2008, and 2011 [8, 43, 44]. Each survey involved a nationally representative and multi-ethnic (black, white, coloured, and Indian) sample of adolescents from grade 8 to 11 in public secondary schools. Only the participants between the ages of 11 and 19 years were included in the analysis as this age range indicates adolescence.

### Procedure

A two-stage cluster sampling was used to recruit participants for the surveys. In stage one, schools were stratified based on the country’s nine provinces. In each province, twenty-three public schools were selected with a probability proportional to student size, i.e. larger schools have a greater probability to be selected (schools with an enrolment of more than 25 learners per grade were considered large, those with less than 25 learners per grade were small). In stage two, classes from grades 8 to grade 11 were selected using systematic equal probability sampling of classes from each selected school. All learners in the selected classes were eligible to participate and complete the survey in their classrooms. The YRBS measured sociodemographics characters and different types of risk behaviours in adolescents, including behaviours related to infectious disease (e.g. sexual activity), chronic disease (e.g. physical activity), injury and trauma (e.g. violence), and mental health (e.g. substance use). For the purpose of this study, we extracted data on participants’ sociodemographic characteristics and behaviours related to injury and trauma, specifically, exposure to violence victimisation and involvement in violence perpetration.

### Measures

#### Sociodemographics

Data were collected on participants’ gender (1 = girl, 0 = boy), age, grade (grade 8 to 11), race (black, coloured,^1^ Indian, white, other), monthly allowance^2^ (‘don’t get any spending money’ to ‘more than R50’), academic performance (‘mostly F’s’ to ‘mostly A’s’), and parental absence (‘I don’t have a father (mother)/my father (mother) is dead’). Parental absence was further coded into three binary variables (1 = father absence, mother absence, or both parents absence, 0 = no absence). The higher scores on the scale variables indicate higher levels of the construct, except for the binary variables of gender and parental absence. Dummy variables were created for race. The majority of adolescents indicated themselves as black and were treated as the reference group in the analyses.

#### Violence victimisation

Participants reported their experiences on six types of indirect and direct victimisation:

*Feeling unsafe in and around school*. Participants were asked to indicate how often they missed school in the past 30 days because they felt they would be unsafe *in* school, and because they felt they would be unsafe on their way *to* and *from* school. The responses for these two items were 1 (0 days) to 5 (6 or more days).

*Been threatened by a weapon at school.* Participants reported the frequency of being threatened by someone with a gun, knife, panga, or kierrie at school in the past 6 months. The responses were 1 (Never) to 5 (Very often).

*Been bullied*. Participants indicated the one way in which they were bullied the most in the past 30 days from the following categories – name calling, physical assault, false rumours, discrimination due to race, discrimination due to religion, sexual jokes/remarks, and others.

*Dating violence victimisation*. Participants reported whether they have ever been hit, smacked, or physically hurt by boy/girlfriend in the past 6 months. The responses were 1 (Yes) and 0 (No).

*Sexual violence victimisation*: participants reported whether they have ever been physically forced to have sex. The responses were 1 (Yes) and 0 (No).

#### Violence perpetration

Participants reported their experiences on eight types of involvement in violence:

*Carry a weapon*. Participants reported the frequency of them carrying a weapon, a gun, a knife in the past 30 days. In addition, they were asked to indicate the frequency of carrying a weapon in school in the past 30 days. The responses for these four items were 1 (Never) to 5 (Very often).

*Threaten others with a weapon*. Participants reported the frequency of them threatening someone with a weapon in school in the past 6 months. The responses were 1 (Never) to 5 (Very often).

*Gang membership*. Participants indicated whether they have been a member of a gang in the past 6 months. The responses were 1 (Yes) and 0 (No).

*Dating violence perpetration*. Participants reported whether they have ever hit, smacked, or physically hurt their boy/girlfriend in the past 6 months. The responses were 1 (Yes) and 0 (No).

*Sexual violence perpetration*. Participants reported whether they have ever physically forced someone to have sex. The responses were 1 (Yes) and 0 (No).

### Data analysis

Data were analysed using SPSS version 23. To obtain insight into the sample characteristics, we first conducted descriptive analyses to investigate the prevalence of each violence typology differentiated by sociodemographic characters for the overall sample and for each survey year. The frequency data were weighted to account for province size and non-response. Weights were post-stratified by grade and gender, so that the weighted counts of participants in each grade and gender combination match the provincial population proportions. Participants in highly populated provinces had higher weights than that from less populated provinces to ensure each province was represented equally in the sample. Moreover, intercorrelations were established between all the variables using point-biserial correlation tests at α = .01.

Next, binary logistic regression analyses were conducted to examine the associations of sociodemographic factors (gender, age, race, and monthly allowance, academic performance, and parental absence) with each violence typology. Since the regression analyses were done separately for the four violence typologies, we applied Bonferroni correction by using a lower criterion for significance (α = .01) to minimise the potential for Type I error [45]. Lastly, trend analysis of complex survey data was performed to investigate the trends in the four violence typologies across the three different survey years (2002, 2008, 2011), following the protocol on Conducting Trend Analysis of YRBS data published by the US Centre for Disease Control and Prevention [46].

We assigned participants into four distinct groups, namely, non-involvement (NI) in victimisation and perpetration, victim only (V), perpetrator only (P), and victim-perpetrator (VP). To do so, the items for violence victimisation and perpetration were dichotomised, so that 0 reflects the participants who did not experience or involve in the type of victimisation/perpetration, whereas 1 reflects the ones who did, regardless the frequency of such experience. This resulted in a possible range of 0–6 for the number of victimisations; and a range of 0–8 for the number of perpetrations. Participants in the V group were the ones who had exposure to one or more victimisations, and the ones who perpetrated one or more violence-related behaviours belong to the P group. Participants in NI did not have any victimisation and perpetration histories (i.e., they scored zero for both victimisation and perpetration). Participants who were in the VP group reported at least one exposure to victimisation and at least one violence-related behaviour.

## Results

### Violence typologies: Descriptives

Table 1 shows the proportions of the adolescents in each violence typology by sociodemographics. The total sample consisted of *n* = 30,007 adolescents (boy: 46.9%, girls: 53.1%; *M* age = 16 years, *SD* = .06). The resulting violence subgroups for the total sample consisted of adolescents who had non-involvement in both victimisation and perpetration (NI, *n* = 8030, 30.8%), victims only (V, *n* = 8217, 29.8%), perpetrators only (P, *n* = 2504, 9.0%), and victim-perpetrators (VP, *n* = 7776, 24.6%). As shown in Table 1, girls disproportionately represented NI (59.0%) and V groups (65.0%), whereas boys disproportionately represented P (67.0%) and VP groups (58.3%). The majority of adolescents in each violence subgroup were black Africans (78.5–84.7%). The majority of adolescents with absent father were in V group (12.4%). The majority with absent mother were in VP group (4.1%), so were the majority with both parents absent (2.6%). Adolescents in NI and V groups had the highest academic performance (*M* = 4.0, *SD* = 0.0). Adolescents in the P group had the highest amount of pocket money (*M* = 3.98, *SD* = 0.1).

**Table 1:** Proportion of South African Adolescents in Each Violence Typology by Sociodemographics

	2002				2008				2011				All			
Sociodemographics	NI (*N* = 2148) 24.6%	V (*N* = 2666) 33.6%	P (*N* = 688) 8.0%	VP(*N* = 2603) 30.0%	NI (*N* = 2666) 27.5%	V (*N* = 2821) 28.7%	P (*N* = 892) 8.9%	VP(*N* = 2861) 31.5%	NI (*N* = 3216) 33.1%	V (2729) 28.6%	P (*N* = 924) 9.3%	VP (*N* = 2312) 22.5%	NI (*N* = 8030) 30.8%	V (*N* = 8217) 29.8%	P(*N* = 2504) 9.0%	VP(*N* = 7776) 24.6%
Gender																
Boy	36,0%	31,9%	66,7%	52,7%	41,5%	39,9%	60,9%	56,9%	42,3%	35,9%	67,3%	60,9%	41,0%	35,0%	67,0%	58,3%
Girl	64,0%	68,1%	33,3%	47,3%	58,5%	60,1%	39,1%	43,1%	57,7%	64,1%	32,7%	39,1%	59,0%	65,0%	33,0%	41,7%
Age (M and SD)	15.77(0.1)	15.95(0.1)	16.08(0.1)	16.08(0.1)	16.04(0.1)	16.12(0.1)	16.02(0.1)	16.41(0.1)	15.99(0.1)	16.07(0.1)	16.00(0.1)	16.36(0.1)	15.95(0.1)	16.04(0.1)	16.02(0.1)	16.28(0.1)
Race																
Black	78,5%	77,9%	73,7%	82,9%	82,6%	80,6%	78,9%	81,9%	84,5%	84,5%	79,8%	85,7%	83,2%	82,6%	78,5%	84,7%
Mixed heritage	8,7%	10,7%	9,9%	8,9%	7,4%	10,8%	11,3%	11,3%	5,8%	7,8%	8,6%	8,1%	6,4%	8,7%	8,9%	8,5%
Indian	1,4%	0,9%	2,2%	1,2%	1,8%	1,3%	2,3%	1,6%	2,5%	1,3%	2,3%	1,6%	2,3%	1,2%	2,3%	1,5%
White	10,9%	9,2%	12,6%	6,0%	7,9%	6,9%	6,7%	4,7%	6,2%	5,3%	8,5%	3,6%	7,2%	6,4%	9,3%	4,4%
Other	0,5%	1,3%	1,6%	1,0%	0,3%	0,4%	0,8%	0,5%	1,0%	1,2%	0,9%	1,0%	0,9%	1,2%	1,0%	1,0%
Parental absence																
Father absence	15,0%	17,0%	13,7%	15,4%	8,4%	10,0%	9,5%	7,5%	11,2%	10,4%	9,0%	9,5%	11,3%	12,4%	10,5%	10,8%
Mother absence	2,6%	2,8%	4,0%	3,9%	2,5%	3,2%	2,4%	4,1%	3,7%	4,4%	3,0%	4,3%	3,0%	3,5%	3,0%	4,1%
Both parents absence	1,0%	1,5%	1,0%	1,8%	2,5%	2,2%	2,5%	2,4%	2,0%	2,8%	2,1%	3,7%	1,9%	2,2%	1,9%	2,6%
Academic performance (M and SD)	4.21(0.1)	4.15(0.1)	4.16(0.1)	4.17(0.0)	4.08(0.1)	4.01(0.1)	3.94(0.1)	4.02(0.1)	3.93(0.1)	3.89(0.1)	3.83(0.1)	3.83(0.1)	4.00(0.0)	4.00(0.0)	3.91(0.1)	3.94(0.0)
Monthly allowance (M and SD)	3.99(0.2)	3.93(0.1)	3.93(0.2)	3.92(0.1)	3.64(0.1)	3.55(0.1)	3.96(0.2)	3.35(0.1)	3.76(0.1)	3.65(0.1)	4.00(0.2)	3.51(0.1)	3.80(0.1)	3.72(0.1)	3.98(0.1)	3.62(0.1)

*Note*. *NI* non-involvement, *V* victims only, *P* perpetrators only, *VP* victim-perpetrators

### Associations between Sociodemographics and violence typologies

Table 2 shows the intercorrelations between all variables. Being a girl was correlated with NI (*r* = .084, *p* < .01) and V (*r* = .146, *p* < .01), whereas being a boy was correlated with P (*r* = −.118, *p* < .01) and VP (*r* = −.150, *p* < .01). Older age was associated with VP (*r* = .079, *p* < .01). Black adolescents were associated with VP (*r* = .030, *p* < .01). Adolescents of mixed heritage were associated with V (*r* = .029, *p* < .01). Indian (*r* = .043, *p* < .01) and white adolescents (*r* = .021, *p* < .01) were associated with NI; they were also associated with P (*r* = .051, *p* < .01; *r* = .024, *p* < .01, respectively). Father absence was associated with V (*r* = .021, *p* < .01). Not having absent mother (*r* = −.016, *p* < .01) was associated with NI, whereas mother absence was associated with VP (*r* = .020, *p* < .01). Both parents absence was associated with VP (*r* = .017, *p* < .01). Higher academic performance was associated with NI (*r* = .017, *p* < .01). Higher monthly allowance was associated with NI (*r* = .015, *p* < .01) and P (*r* = .044, *p* < .01), whereas lower monthly allowance was associated with VP (*r* = −.018, *p* < .01).

**Table 2:** Intercorrelations between Sociodemographics and Violence Typologies in South African Adolescents

	1	2	3	4	5	6	7	8	9	10	11	12	13	14	15	16
1. Gender	_															
2. Age	−.088^**^	_														
3. Black	−.002	.141^**^	_													
4. Mixed heritage	.006	−.085^**^	−.669^**^	_												
5. Indian	.004	−.083^**^	−.392^**^	−.090^**^	_											
6. White	−.006	−.057^**^	−.442^**^	−.101^**^	−.059^**^	_										
7. Other	−.008	−.005	−.166^**^	−.038^**^	−.022^**^	−.025^**^	_									
8. Father absence	.032^**^	.048^**^	.074^**^	−.030^**^	−.036^**^	−.056^**^	−.008	_								
9. Mother absence	.013	.018^**^	.044^**^	−.024^**^	−.021^**^	−.027^**^	.001	−.069^**^	_							
10. Both parents absence	.003	.038^**^	.049^**^	−.022^**^	−.030^**^	−.033^**^	.007	−.054^**^	−.029^**^	_						
11. Academic performance	.032^**^	−.188^**^	−.040^**^	−.005	.060^**^	.020^**^	.009	−.021^**^	−.016^**^	−.028^**^	_					
12. Monthly allowance	.001	−.067**	−.256**	.103**	.109**	.221**	−0,01	−.032**	−.029**	−.047**	.037**	_				
13. NI	.084**	−.059**	−.005	−.033**	.043**	.021**	−.010	−.001	−.016**	−.014	.017**	.015**	_			
14. V	.146**	−.019**	−.014	.029**	−.029**	.008	.006	.021**	−.000	−.001	−.011	−.015	−.428**	_		
15. P	−.118**	−.013*	−.033**	−.006	.051**	.024**	−.003	−.009	−.008	−.006	−.006	.044**	−.202**	−.216**	_	
16. VP	−.150**	.079**	.030**	.016	−.043**	−.037**	.003	−.013	.020**	.017**	−.008	−.018**	−.424**	−.422**	−.203**	_

** *p* < .01; * *p* < .05

*Note*. *NI* non-involvement, *V* victims only, *P* perpetrators only, *VP* victim-perpetrators

### Binary logistic regression analyses

Significant associations of sociodemographic variables with the four violence typologies are reported here. A full overview of the results is presented in Table 3. *Non-involvement*. Being a girl (OR: 1.47, 99% CI: 1.36–1.58) and having a younger age (OR: 0.94, 99% CI: 0.91–0.96) were associated with higher odds of NI. Black adolescents had higher odds of NI than adolescents of mixed heritage (OR: 0.82, 99% CI: 0.73–0.92). Further, Indian (OR: 1.49, 99% CI: 1.27–1.75) and white adolescents (OR: 1.22, 99% CI: 1.05–1.42) had higher odds of NI than black adolescents. In addition, adolescents who did not have an absent mother had higher odds of NI (OR: 0.78, 99% CI: 0.62–0.97).

**Table 3:** Results of Binary Logistic Regression Analyses on Violence Typologies with Sociodemographics in South African Adolescents

	NI		V		P		VP	
Variables	OR	99% CL	OR	99% CL	OR	99% CL	OR	99% CL
Gender^abcd^	1,47	1.36–1.58	1,90	1.76–2.05	0,42	0.37–0.47	0,51	0.47–0.55
Age^ad^	0,94	0.91–0.96	0,98	0.96–1.01	0,96	0.92–1.00	1,10	1.07–1.13
Black (reference)								
Mixed heritage^ab^	0,82	0.73–0.92	1,22	1.09–1.37	0,91	0.76–1.10	1,06	0.95–1.19
Indian^abcd^	1,49	1.27–1.75	0,80	0.67–0.96	1,70	1.35–2.14	0,61	0.50–0.75
White^acd^	1,22	1.05–1.42	1,13	0.97–1.31	1,24	0.99–1.55	0,67	0.57–0.80
Other	0,80	0.51–1.25	1,32	0.87–2.01	0,87	0.42–1.79	0,94	0.60–1.47
Monthly allowance^bc^	1,00	0.99–1.02	0,99	0.97–1.00	1,05	1.03–1.08	1,00	0.98–1.01
Academic performance	1,01	0.98–1.04	0,97	0.95–1.00	0,98	0.93–1.02	1,02	0.99–1.05
Father absence	1,01	0.89–1.14	1,10	0.98–1.24	1,00	0.82–1.21	0,90	0.79–1.02
Mother absence^a^	0,78	0.62–0.97	1,07	0.87–1.32	0,91	0.64–1.30	1,20	0.97–1.47
Both parents absence	0,89	0.68–1.17	0,88	0.67–1.15	1,06	0.69–1.62	1,22	0.94–1.57

^a^significant variables for NI

^b^significant variables for V

^c^significant variables for P

^d^significant variables for VP

*Note*. Significance is at the 0.01 level. *NI* non-involvement, *V* victims only, *P* perpetrators only, *VP* victim-perpetrators

#### Victims only

Being a girl (OR: 1.90, 99% CI: 1.76–2.05) was associated with higher odds of only being a victim. Adolescents of mixed heritage had higher odds of being victimised only than black adolescents (OR: 1.22, 99% CI: 1.09–1.37). Black adolescents had higher odds of being victimised only than Indian adolescents (OR: 0.80, 99% CI: 0.67–0.96). Adolescents who had lower amount of monthly allowance had higher odds of being victimised only (OR: 0.99, 99% CI: 0.97–1.00).

#### Perpetrators only

Being a boy (OR: 0.42, 99% CI: 0.37–0.47) was associated with higher odds of only being a perpetrator of violence. Indian (OR: 1.70, 99% CI: 1.35–2.14) and white adolescents (OR: 1.24, 99% CI: 0.99–1.55) had higher odds of only perpetrating violence than black adolescents. Higher monthly allowance was associated with higher odds of only perpetrating violence (OR: 1.05, 99% CI: 1.03–1.08).

#### Victim-perpetrators

Being a boy (OR: 0.51, 99% CI: 0.47–0.55) and having an older age (OR: 1.10, 99% CI: 1.07–1.13) were associated with higher odds of being a VP. Black adolescents had higher odds of being VP than Indian adolescents (OR: 0.61, 99% CI: 0.50–0.75) and white adolescents (OR: 0.67, 99% CI: 0.57–0.80).

### Trend analysis

#### Non-involvement

The overall prevalence of adolescents who had no victimisation and perpetration experiences was 30.8%, (95% CI: 0.29–0.32). The prevalence of these adolescents increased from 24.6%, (95% CI: 0.23–0.27) in 2002, to 27.5%, (95% CI: 0.25–0.30) in 2008, and to 33.1%, (95% CI: 0.31–0.35) in 2011, indicating a significant increasing trend from 2002 to 2011 (*p* < .001). Thus, involvement in violence (either victimisation or perpetration) declined over these years.

#### Victims only

The overall prevalence of adolescents who were victims only and had at least one victimisation experience was 29.8%, (95% CI: 0.28–0.31). The prevalence of these adolescents decreased from 33.6% (95% CI: 0.31–0.36) in 2002, to 28.7% (95% CI: 0.27–0.30) in 2008, and to 28.6% (95% CI: 0.27–0.30) in 2011, which showed a significant decreasing trend from 2002 to 2011 (*p* < .01).

#### Perpetrators only

The overall prevalence of adolescents who were perpetrators only and perpetrated at least one violence-related act was 9.0%, (95% CI: 0.08–0.10). There were no significant changes in the prevalence of these adolescents across 2002 (8.0, 95% CI: 0.07–0.09), 2008 (8.9, 95% CI: CI: 0.08–0.10), and 2011 (9.3, 95% CI: 0.09–0.10) (*p* > .05). Thus, rates of violence perpetration among adolescents aged 11–19 remained relatively stable over these years.

#### Victim-perpetrators

The overall prevalence of adolescents who had at least one victimisation and perpetration experience was 24.6%, (95% CI: 0.23–0.26). The prevalence of these adolescents decreased from 30.0%, (95% CI: 0.27–0.34) in 2002 and 31.5%, (95% CI: 0.29–0.34) in 2008, to 22.5%, (95% CI: 0.21–0.24) in 2011, which showed a significant decreasing trend from 2002 to 2011 (*p* < .001).

## Discussion

This study provided an overview of the national prevalence of the school adolescents in South Africa who were non-involved in both violence victimisation and perpetration, victims only, perpetrators only, and victim-perpetrators in 2002, 2008, and 2011, as well as the trend changes in the prevalence of these four violence typologies across the survey years. Moreover, the associations of demographics, academic performance, pocket money, and parental absence with the violence typologies were examined to understand the unique correlates of each violence subgroup.

Approximately a third of the adolescents in the whole sample were classified as victims only, and another third had non-involvement in both victimisation and perpetration. When the prevalences were examined by each survey year, the proportion of adolescents who were victims only significantly decreased from 2002 to 2011, whereas the adolescents who hadnone-involvement significantly increased from 2002 to 2011. These trend changes reflect a positive social transition in the post-Apartheid era in South Africa, where political violence of discrimination against non-whites (such as forced removal and brutal physical assault) has subsided since the abolishment of Apartheid in 1994. In the years following this event, new legislation and policies have been enforced to prevent crime and improve criminal justice functioning in democratic South Africa [47]. These changes are likely to have contributed to the overall reduction of violence victimisation among young people.

Moreover, the lowest proportion of adolescents in the whole sample were perpetrators only, with less than 10% were found in each survey year and the prevalence remained stable across 2002 to 2011. Importantly, we found that a substantial proportion of adolescents (almost a quarter) were both a victim and a perpetrator of violence. This result is in line with other studies [5, 33, 34, 48] that demonstrated that victimisation and perpetration experiences can overlap. In South Africa, where high levels of interpersonal violence still exist, such violence is marked by contextual features, such as poverty, unemployment, gender inequality, exposure to abuse in childhood, and access to firearms [49]. Both victims and perpetrators may share similar contextual features that put them at risk for both forms of violence. Furthermore, a review has found that exposure to different forms of violence such as sexual, emotional, and physical abuse, and even less severe forms of physical punishment and harsh parenting may have adverse long term impact, and increase the likelihood of later violence perpetration among youth [39]*.* One possible mechanism of such effects may be due to the social learning effects of violence through direct observation and socialisation processes [50], and that adolescents acquired violent behaviours through learning and internalising it as the “norm” to solve problems, particularly in South Africa where there was a history of violence [51]. In addition, the risk for violent behaviours can be compounded by the propensity for risk taking behaviours and vulnerability to emotion dysregulation during the developmental phase of adolescence [52].

Further, our results showed that each violence typology was associated with different sociodemographic factors. We found that a lower amount of monthly pocket money put adolescents at risk for victimisation. This result corroborates with studies that showed that compared to adolescents from higher socio-economic environments, violence exposure among adolescents from low socioeconomic settings is normative, and they experience violence either directly or indirectly on a daily basis [4, 53]. Conversely, we found that higher amount of pocket money put adolescents at risk for perpetration of violence towards others. This association might be related to the purchase of the highly available alcohol, drugs, and weapons in many South African communities [54, 55], which may in turn promote violent behaviours.

Moreover, we found that boys and girls are at risk for different violence experiences. Being male was a significant predictor for having only perpetrated violence. Adolescents are in a developmental period where they are inclined to be sensation seeking and risk taking [56], and boys in particular, are at risk for externalising problems such as aggression, aggressive fantasies, conduct problems, and higher levels of delinquency [9, 18, 57]. Our results also indicated that boys were more likely to be victim-perpetrators than girls, which is in line with other studies that showed correlations between violence victimisation and violence perpetration in adolescents [34, 35]. This may be due to the socialisation differences between boys and girls in South Africa, in which boys are more likely to spend time in their neighbourhoods and to be approached by or be involved in gangs [4, 58], putting them at risk for both the exposure to and the conduct of violent activities. Furthermore, boys are more likely than girls to engage in high-risk behaviours such as alcohol and drug use [59, 60], which can expose them to additional high risk situations, including greater exposure to potential offenders and an elevated risk for both violence victimisation and perpetration [61, 62].

On the other hand, being a girl was a significant predictor for non-involvement in both victimisation and perpetration. This could be explained in light of findings that girls are more prone to internalising symptoms such as anxiety, depression, and perceived stress during adolescence [7, 9, 63, 64], and may thus experience less tendencies to engage in externalising behaviours such as violence perpetration. Further, girls in our study were also associated with having only victimisation experiences. Although some studies have established that boys are at higher risk than girls for victimisation in different contexts such as school and community [7, 53] as well as poly-victimisation [4, 13], the current finding indicates the opposite and may highlight the vulnerability of girls to victimisation. According to a national survey in South Africa, females – both girls and adult women – are subjected to high levels of physical violence [65] and are more likely to be victimised by certain types of violence than boys, such as sexual assault, rape, and dating violence [14, 66].

Another important finding in our study is that adolescents who did not have an absent mother in the household (absent due to divorce or death) was associated with non-involvement in victimisation and perpetration, suggesting that the presence of maternal support and supervision may have positive implications on an adolescent’s life experiences and adjustment. Indeed, functioning parent-child relationship such as family support has been found to be a protective factor that may lower the risk for violence perpetration in boys as well as in adolescents who had exposure to community violence [67]. Similarly, high mother-child relationship quality such as adequate involvement and supervision, and parenting that satisfies the child’s needs for security, exploration, and autonomy, can protect against internalising (e.g. anxiety, depression) and externalising (e.g. aggressive behaviours) adjustment difficulties in youth [68–72]. Conversely, higher levels of maternal rejection are associated with greater behaviour problems such as aggression [73, 74]. Moreover, there is evidence that single fathers have weaker interpersonal and affective bonds with their children, and exhibit less supervision and monitoring [75], compounding the possible effects of mother absence. However, the current results prohibit us from drawing firm conclusions, and these interpretations thus remain speculative, and future research is needed to understand the exact underlying processes of family influence on adolescents’ experiences of violence victimisation and perpetration.

### Limitations and recommendations

Although this study provided an overview of the violence trend across three survey years (2002, 2008, and 2011) among a nationally representative sample of South African adolescents, few limitations should be noted. First, the results of the study are limited to the time period between 2002 and 2011 as a new YRBS has not been conducted in South Africa since 2011. The data consisted of three cross-sectional samples and thus the causal direction of the associations between violence typologies and their sociodemographic correlates cannot be established. Longitudinal studies are necessary to investigate the causality of these associations, as well as the possible moderating variables. Moreover, the intercorrelations between variables are weak in this study. Although they are statistically significant, it may be due to the nature of a large sample and thus may limit the practical implications of these associations. In addition, the reasons for parental absence in life were not explicitly accounted for in this study and require further exploration. For example, a parent may be absent due to work, divorce, or death, and each may have differential impact and underlying mechanisms on adolescents’ experiences of violence. Lastly, data were collected in schools and may not be fully representative for adolescents who do not attend school. In South Africa, approximately 8 % of adolescents are secondary school drop-outs [76]. Due to inadequately developed life and social skills, these adolescents are more likely to engage in high risk activities, including violence [77]. Future research may draw attention on this unique population to obtain a more comprehensive understanding of the violence profiles among different subgroups of adolescents.

## Conclusions

This current study showed a significant reduction in the prevalence of adolescent victims of violence as well as victim-perpetrators between 2002 and 2011 in South Africa, suggesting that the crime and violence reduction strategies implemented in the post-Apartheid era had a positive effect on adolescents’ violence victimisation experiences. Our study sheds light on the sociodemographic determinants of violence that are beyond the scope of the police and courts, such as income inequality, ethnicity, gender inequality, and compromised parenting [49]. In addition, our results offer insights that presence of mother in an adolescent’s life may bring positive influences on the child’s development. Interventions may consider strengthening family relations, particularly between mother and child, to protect adolescents from the experiences of violence victimisation and violence perpetration. Since studies have consistently established that family connectedness is associated with positive youth developmental outcomes such as lower levels of violence victimisation and aggression [67–72], there is an opportunity for families with mother absence to involve extended family members to extend the support and supervision that may be needed to promote safety and positive behavioural adjustment among adolescents. In conclusion, regular monitoring of the prevalence of violence victimisation and violence perpetration as well as the associated correlates may help develop targeted youth interventions in South Africa. Given the heterogeneity of violence profiles and the unique correlates of each violence subgroup as highlighted in our study, one-size-fits-all violence interventions may be ineffective and tailored interventions are needed for adolescents with differed risks to violence exposure and violence perpetration.

## Data Availability

The datasets used and/or analysed during the current study are available from the corresponding author on reasonable request.

## Abbreviations

NI: Non-involvement (in victimisation and perpetration)
P: Perpetrators only
V: Victims only
VP: Victim-perpetrators
YRBS: Youth Risk Behaviour Survey

## Footnotes

1 A South African term referred to people of mixed heritage. This term is officially used in statistical publications and census data in South Africa.

2 The amount of monthly allowance indicated in the three surveys (2002, 2008, 2011) increased around 30% over the survey years due to inflation.

## References

[1] Organization WH (2019). Maternal, newborn, child and adolescent health: adolescent health epidemiology.

[2] Zimmerman GM, Posick C (2016). Risk factors for and behavioural consequences of direct versus indirect exposure to violence. Am J Public Health.

[3] Krug E, Dahlberg L, Mercy J, Zwi A, Lozano R (2002). World report on violence and health.

[4] Kaminer D, du Plessis B, Hardy A, Benjamin A (2013). Exposure to violence across multiple sites among young South African adolescents. Peace Conflict.

[5] Shields N, Nadasen K, Pierce L (2009). A comparison of the effects of witnessing community violence and direct victimisation among children in Cape Town, South Africa. J Interpers Violence.

[6] Leoschut L, Kafaar Z (2017). The frequency and predictors of poly-victimisation of south African children and the role of schools in its prevention. Psychol Health Med.

[7] Sui X, Massar K, Kessels LTE, Reddy PS, Ruiter RAC, Sanders-Phillips K. Violence exposure in south African adolescents: differential and cumulative effects on psychological functioning. J Interpers Violence. 2018:1–27. 10.1177/0886260518788363 PMC8064538 30024299

[8] Reddy SP, James S, Sewpaul R, Sifunda S, Ellahebokus A, Kambaran NS (2013). Umthente uhlaba usamila: the 3rd South African national youth risk behaviour survey 2011.

[9] Mrug S, Loosier PS, Windle M (2008). Violence exposure across multiple contexts: individual and joint effects on adjustment. Am J Orthop.

[10] Botticello AL (2009). A multilevel analysis of gender differences in psychological distress over time. J Res Adolesc.

[11] Das-Munshi J, Lund C, Mathews C, Clark C, Rothon C, Stansfeld S (2016). Mental health inequalities in adolescents growing up in post-apartheid South Africa: cross-sectional survey, SHaW study. PLoS One.

[12] Bronfenbrenner U (1979). The ecology of human development: experiments by nature and design.

[13] Finkelhor D, Ormrod RK, Turner HA (2007). Poly-victimisation: a neglected component in child victimisation. Child Abuse Negl.

[14] Finkelhor D (2009). Children’s exposure to violence: a comprehensive national survey.

[15] Leiner M, Dwivedi AK, Villanos MT, Singh N, Blunk D, Peinado J (2014). Psychosocial profile of bullies, victims, and bully-victims: a cross-sectional study. Front Pediatr.

[16] Sharma MK, Marimuthu P (2014). Prevalence and psychosocial factors of aggression among youth. Indian J Psychol Med.

[17] Hong JS, Kim DH, Hunter SC (2017). Applying the social–ecological framework to explore bully–victim subgroups in South Korean schools. Psychology of Violence.

[18] Reingle JM, Maldonado-Molina MM (2012). Victimisation and violent offending: an assessment of the victim-offender overlap among native American adolescents and young adults. Int Criminal Justice Rev.

[19] Reyes HLM, Foshee VA, Chen MS, Ennett ST (2017). Patterns of dating violence victimisation and perpetration among Latino youth. J Youth Adolescence.

[20] Gevers A, Flisher A, Ward C, van der Merwe A, Dawes A (2012). School-based youth violence prevention interventions. Youth violence: sources and solutions in South Africa.

[21] Bernat DH, Oakes JM, Pettingell SL, Resnick M (2012). Risk and direct protective factors for youth violence: results from the national longitudinal study of adolescent health. Am J Prev Med.

[22] Henry DB, Tolan PH, Gorman-Smith D, Schoeny ME (2012). Risk and direct protective factors for youth violence: results from the centers for disease control and prevention’s multisite violence prevention project. Am J Prev Med.

[23] Herrenkohl TI, Huang B, Tajima EA, Whitney SD (2003). Examining the link between child abuse and youth violence: an analysis of mediating mechanisms. J Interpersonal Violence.

[24] van der Merwe A, Dawes A (2007). Youth violence: a review of risk factors, causal pathways and effective intervention. J Child Adolesc Ment Health.

[25] Hammig B, Jozkowski K (2013). Academic achievement, violent victimisation, and bullying among U.S. high school students. J Interpers Violence.

[26] Meintjes H, Hall K, Marera D, Boulle A (2010). Orphans of the AIDS epidemic? The extent, nature and circumstances of child-headed households in South Africa. AIDS Care.

[27] Morantz G, Cole D, Vreeman R, Ayaya S, Ayuku D, Braitstein P (2013). Child abuse and neglect among orphaned children and youth living in extended families in sub-Saharan Africa: what have we learned from qualitative inquiry?. Vulnerable Children & Youth Studies.

[28] Raymond JM, Zolnikov TR (2018). Aids-affected orphans in sub-saharan africa: a scoping review on outcome differences in rural and urban environments. AIDS and Behaviour.

[29] Ellonen N, Salmi V (2011). Poly-victimisation as a life condition: correlates of poly-victimisation among Finnish children. J Scandinavian Studies Criminol Crime Prev.

[30] Turner HA, Finkelhor D, Hamby S, Henly M (2017). Victimisation and adversity among children experiencing war-related parental absence or deployment in a nationally representative US sample. Child Abuse Negl.

[31] Dekel B, Abrahams N, Andipatin M (2018). Exploring adverse parent-child relationships from the perspective of convicted child murderers: a south African qualitative study. PLoS One.

[32] Harper CC, McLanahan SS (2004). Father absence and youth incarceration. J Res Adolesc.

[33] Whiteside LK, Ranney ML, Chermack ST, Zimmerman MA, Cunningham RM, Walton MA (2013). The overlap of youth violence among aggressive adolescents with past-year alcohol use-a latent class analysis: aggression and victimisation in peer and dating violence in an inner city emergency department sample. J Studies Alcohol Drugs.

[34] Choe DE, Zimmerman MA, Devnarain B (2012). Youth violence in South Africa: exposure, attitudes, and resilience in Zulu adolescents. Violence Vict.

[35] Jeong S, Davis J, Han Y (2015). Who becomes more violent among Korean adolescents? Consequences of victimisation in school. Crim Behav Ment Health.

[36] Voisin D, Hong J (2012). A meditational model linking witnessing intimate partner violence and bullying behaviours and victimisation among youth. Educ Psychol Rev.

[37] Liu W, Mumford EA, Taylor BG (2018). The relationship between parents’ intimate partner victimisation and youths’ adolescent relationship abuse. Journal of Youth and Adolescence.

[38] Kaukinen C, Buchanan L, Gover A (2015). Child abuse and the experience of violence in college dating relationships: examining the moderating effect of gender and race. J Fam Violence.

[39] Maas C, Herrenkohl TI, Sousa C (2008). Review of research on child maltreatment and violence in youth. Trauma Violence Abuse.

[40] Russell PL, Nurius PS, Herting JR, Walsh E, Thompson EA (2010). Violent victimisation and perpetration: joint and distinctive implications for adolescent development. Vict Offenders.

[41] Georgiou SN, Stavrinides P (2008). Bullies, victims and bully-victims: psychosocial profiles and attribution styles. Sch Psychol Int.

[42] Haynie DL, Farhat T, Brooks-Russell A, Wang J, Barbieri B, Iannotti RJ (2013). Dating violence perpetration and victimisation among US adolescents: prevalence, patterns, and associations with health complaints and substance use. J Adolesc Health.

[43] Reddy SP, Panday S, Swart D, Jinabhai CC, Amosun SL, James S (2003). Umthenthe Uhlaba Usamila – the south African youth risk behaviour survey 2002.

[44] Reddy SP, James S, Sewpaul R, Koopman F, Funani NI, Sifunda S (2010). Umthente Uhlaba Usamila – the south African youth risk behaviour survey 2008.

[45] Field A. Discopering statistics using SPSS. 3rd ed. Thousand Oaks: SAGE Publications; 2009.

[46] Control CfD. Conducting Trend Analysis of YRBS data 2014 [Available from: http://www.cdc.gov/healthyyouth/yrbs/pdf/yrbs_conducting_trend analyses.pdf.

[47] Du Plessis A, Louw A (2005). Crime and crime prevention in South Africa: 10 years after. Can J Criminol Crim Justice.

[48] Swahn MH, Gressard L, Palmier JB, Kasirye R, Lynch C, Yao H (2012). Serious violence victimisation and perpetration among youth living in the slums of Kampala, Uganda. West J Emerg Med.

[49] Seedat M, Van Niekerk A, Suffla S, Ratele K (2014). Psychological research and South Africa’s violence prevention responses. S Afr J Psychol.

[50] Bandura A (1977). Self-efficacy: toward a unifying theory of behavioural change. Psychol Rev.

[51] van der Merwe A, Dawes A, Ward CL, Ward CL, van der Merwe A, Dawes A (2012). The development of youth violence: an ecological understanding. Youth violence: sources and solutions.

[52] Bell CC, McBride DF (2010). Affect regulation and prevention of risky behaviours. JAMA.

[53] Kaminer D, Hardy A, Heath K, Mosdell J, Bawa U (2013). Gender patterns in the contribution of different types of violence to posttraumatic stress symptoms among south African urban youth. Child Abuse Negl.

[54] Burton P, Leoschut L (2013). School violence in South Africa: results of the 2012 national school violence study.

[55] Matzopoulos RG, Thompson ML, Myers JE (2014). Firearm and nonfirearm homicide in 5 south African cities: a retrospective population-based study. Am J Public Health.

[56] Steinberg L (2007). Risk taking in adolescence: new perspectives from brain and behavioural science. Curr Dir Psychol Sci.

[57] Duke AA, Smith KZ, Oberleitner LS, Westphal A, McKee SA (2018). Alcohol, drugs, and violence: a meta-meta-analysis. Psychol Violence.

[58] Cooper A, Ward CL, Ward CL, AvdM AD (2012). Intervening with Youth in Gangs. Youth violence: sources and solutions.

[59] Croisant SP, Haque Laz T, Rahman M, Berenson AB (2013). Gender differences in risk behaviours among high school youth. Global Advances in Health and Medicine.

[60] Mennis J, Mason MJ (2012). Social and geographic contexts of adolescent substance use: the moderating effects of age and gender. Soc Networks.

[61] Mrug S, Windle M (2009). Bidirectional influences of violence exposure and adjustment in early adolescence: externalizing behaviours and school connectedness. J Abnorm Child Psychol.

[62] Parker EM, Bradshaw CP (2015). Teen dating violence victimisation and patterns of substance use among high school students. J Adolesc Health.

[63] Hilt LM, Cha CB, Nolen-Hoeksema S (2008). Nonsuicidal self-injury in young adolescent girls: moderators of the distress-function relationship. J Consult Clin Psychol.

[64] Slone M, Mayer Y (2015). Gender differences in mental health consequences of exposure to political violence among Israeli adolescents. Child Youth Serv Rev.

[65] Africa SS (2016). South Africa Demographic and Health Survey 2016.

[66] Duque LF, Montoya NE, Restrepo A (2011). Violence witnessing, perpetrating and victimisation in medellin, Colombia: a random population survey. BMC Public Health.

[67] Massarwi AA (2017). The correlation between exposure to neighborhood violence and perpetration of moderate physical violence among Arab-Palestinian youth: can it be moderated by parent-child support and gender?. Am J Orthop.

[68] Al-Yagon M (2015). Externalizing and internalizing behaviours among adolescents with learning disabilities: contribution of adolescents’ attachment to mothers and negative affect. J Child Fam Stud.

[69] Kliewer W, Cunningham JN, Diehl R, Parrish KA, Walker JM, Atiyeh C (2004). Violence exposure and adjustment in inner-city youth: child and caregiver emotion regulation skill, caregiver–child relationship quality, and neighborhood cohesion as protective factor. J Clin Child Adolescent Psychol.

[70] McWey LM, Claridge AM, Wojciak AS, Lettenberger KCG (2015). Parent–adolescent relationship quality as an intervening variable on adolescent outcomes among families at risk: dyadic analyses. Family Relations.

[71] Profe W, Wild LG (2017). Mother, father, and grandparent involvement. J Fam Issues.

[72] Villodas MT, Bagner DM, Thompson R (2018). A step beyond maternal depression and child behaviour problems: the role of mother-child aggression. J Clin Child Adolescent Psychol.

[73] Howard K, Martin A, Berlin LJ, Brooks-Gunn J (2011). Early mother-child separation, parenting, and child well-being in early head start families. Attach Hum Dev.

[74] Schiff M, Pat-Horenczyk R, Ziv Y, Brom D. Multiple traumas, maternal depression, mother-child relationship, social support, and young children’s behavioural problems. J Interpers Violence. 2017. p. 1–23. 10.1177/0886260517725738 29294915

[75] Coles RL (2015). Single-father families: a review of the literature. J Fam Theory Rev.

[76] Department of Basic Education (2011). Report on Dropout and Learner Retention Strategy to Portfolio Committee on Education Pretoria, South Africa.

[77] Vayachuta P, Ratana-Ubol A, Soopanyo W (2016). The study of ‘out-of-school’ children and youth situations for developing a lifelong education model for ‘out-of-school’ children and youth. SHS Web of Conferences.

